# Characterization of small ruminant lentivirus A4 subtype isolates and assessment of their pathogenic potential in naturally infected goats

**DOI:** 10.1186/1743-422X-11-65

**Published:** 2014-04-03

**Authors:** Martina Deubelbeiss, Laure Blatti-Cardinaux, Marie-Luise Zahno, Reto Zanoni, Hans-Rudolf Vogt, Horst Posthaus, Giuseppe Bertoni

**Affiliations:** 1 Institute of Virology and Immunology, Vetsuisse Faculty, University of Bern, Bern, Switzerland; 2 Institute of Animal Pathology, Vetsuisse Faculty, University of Bern, Bern, Switzerland

**Keywords:** CAEV, VMV, MVV, Visna, Maedi, SRLV, Virulence, Pathogenicity, Attenuation, Eradication

## Abstract

**Background:**

Small ruminant lentiviruses escaping efficient serological detection are still circulating in Swiss goats in spite of a long eradication campaign that essentially eliminated clinical cases of caprine arthritis encephalitis in the country. This strongly suggests that the circulating viruses are avirulent for goats.

To test this hypothesis, we isolated circulating viruses from naturally infected animals and tested the in vitro and in vivo characteristics of these field isolates.

**Methods:**

Viruses were isolated from primary macrophage cultures. The presence of lentiviruses in the culture supernatants was monitored by reverse transcriptase assay. Isolates were passaged in different cells and their cytopathogenic effects monitored by microscopy. Proviral load was quantified by real-time PCR using customized primer and probes. Statistical analysis comprised Analysis of Variance and Bonferroni Multiple Comparison Test.

**Results:**

The isolated viruses belonged to the small ruminant lentiviruses A4 subtype that appears to be prominent in Switzerland. The 4 isolates replicated very efficiently in macrophages, displaying heterogeneous phenotypes, with two isolates showing a pronounced cytopathogenicity for these cells. By contrast, all 4 isolates had a poor replication capacity in goat and sheep fibroblasts. The proviral loads in the peripheral blood and, in particular, in the mammary gland were surprisingly high compared to previous observations. Nevertheless, these viruses appear to be of low virulence for goats except for the mammary gland were histopathological changes were observed.

**Conclusions:**

Small ruminant lentiviruses continue to circulate in Switzerland despite a long and expensive caprine arthritis encephalitis virus eradication campaign. We isolated 4 of these lentiviruses and confirmed their phylogenetic association with the prominent A4 subtype. The pathological and histopathological analysis of the infected animals supported the hypothesis that these A4 viruses are of low pathogenicity for goats, with, however, a caveat about the potentially detrimental effects on the mammary gland. Moreover, the high proviral load detected indicates that the immune system of the animals cannot control the infection and this, combined with the phenotypic plasticity observed in vitro, strongly argues in favour of a continuous and precise monitoring of these SRLV to avoid the risk of jeopardizing a long eradication campaign.

## Background

Caprine arthritis encephalitis virus (CAEV) and Visna/Maedi virus (VMV) are classified as two distinct species in the genus lentivirus of the Orthoretrovirinae subfamily and were, for many years, thought to be species specific viruses of goats and sheep, respectively. Several members of these virus species, however, efficiently cross the species barrier between goats and sheep; therefore, CAEV (SRLV B) and VMV (SRLV A) are now referred to as small ruminant lentiviruses (SRLV) [1].

SRLV cause persistent infections and may induce clinical disease in about one third of the infected animals following an incubation time of months to years. The course of infection is influenced by host genetic factors, potentially controlling virus entry, intrinsic resistance mechanisms, as well as the quality of the immune response to the infecting virus [2-4]. Viral virulence factors, or lack thereof, are also considered important determinants influencing the outcome of an infection. Indeed, SRLV carrying mutations in their dUTPase encoding gene or, due to natural or engineered mutations lacking intact vif or vpr (tat) open reading frames, show an attenuated phenotype [5-8]. Several mutations, deletions or duplications in the long terminal repeats (LTR) of SRLV were also shown to be associated with virulence, attenuation, or change of tissue tropism of these viruses [9-13].

In the eighties Switzerland started a very successful CAEV eradication program that reduced the seroprevalence in goats from between 60 and 80% to around 1%. As a result, the clinical manifestations of CAEV, which are essentially associated with type B SRLV, completely disappeared from the Swiss goat population.

Unfortunately, however, SRLV “outbreaks” characterized by sudden seroconversions in flocks certified to be free from CAEV for years are regularly detected, jeopardizing the eradication campaign. In recent years we have focused our research on these particular flocks and have shown that SRLV A4 viruses are often involved in these infections. The fact that the serological tools used to monitor the eradication campaign are inefficient at detecting SRLV A4 infected animals suggests that these viruses are most probably endemic, at least in some Swiss regions [14].

The decision of the federal veterinary authority to focus the CAEV eradication campaign only on goats infected with SRLV subtype B viruses emphasizes the importance of establishing the pathogenic potential of SRLV A4 viruses in goats. We have not to date detected any animals with clinical signs related to SRLV infections, even in flocks with high seroprevalence. This is strong evidence that the SRLV A4 subtypes are attenuated viruses. In this work we selected 5 adult, seropositive goats from a large flock with a high SRLV A4 seroprevalence with the aim of characterizing the infecting virus, quantifying the proviral load in blood and different organs of these animals and monitoring potential histopathological lesions induced by this particular SRLV subtype.

## Results

### Virus isolation and cytopathic effects

Virus isolation from PBMC was successful in 4 out of 5 goats. Repeated attempts to isolate virus from goat #1 invariably failed.

The isolated viruses showed different phenotypes when cultured on macrophages, goat synovial membrane cells (GSM) or lamb synovial membrane cells (LSM).

**Figure 1:**
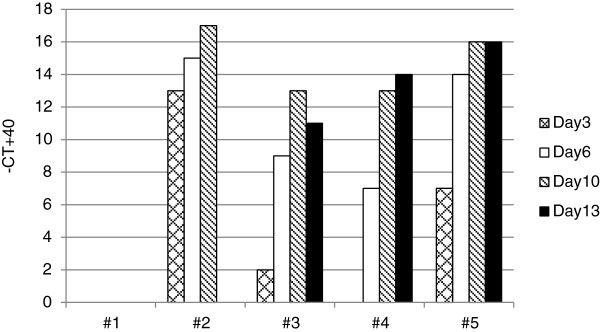
**Virus isolation.** RT activity in the supernatant of macrophage cultures monitored over 13 days. Results are expressed as - CT values + 40 according to the described PERT assay.

In the supernatant of macrophage cultures obtained from goats #2, #3 and #5 a reverse transcriptase (RT) activity was readily detected by PERT-assay starting at day 3 post isolation (p.iso.), while for goat #4 RT activity was present starting at day 6 p.iso. (Figure 1). With the exception of macrophage cultures of goat #2, showing a massive loss of cells between day 10 and 13 p.iso., the RT activity in the supernatants of all macrophage cultures increased till day 10 to 13 p.iso.. The morphology of these cells was indistinguishable from that of control macrophages obtained from certified SRLV free goats. As mentioned above, the only exceptions were the macrophage cultures of goat #2 showing an increasing cytopathic effect that prevented us to measure the RT activity at day 13 p.iso., when essentially all cells were dead.

**Figure 2:**
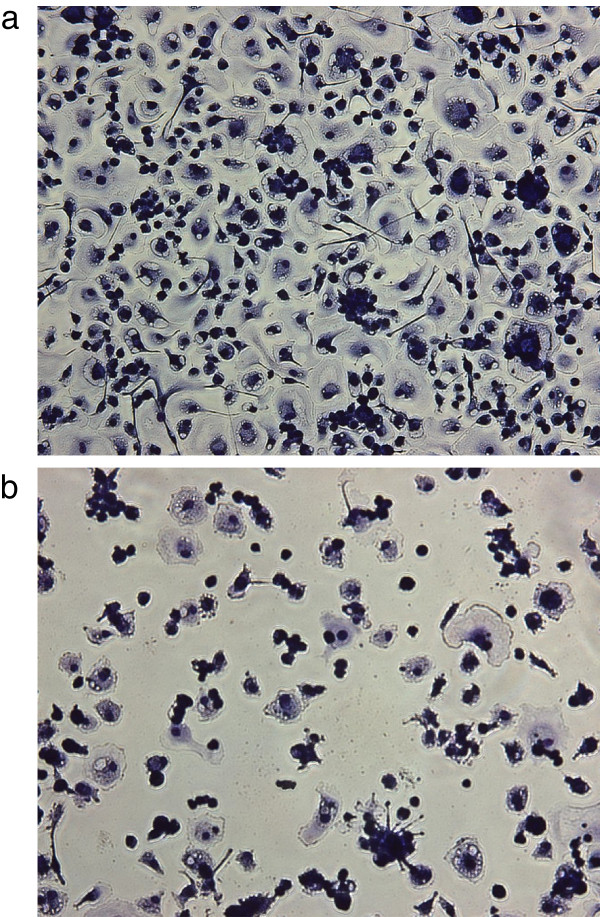
**Cytopathic effect on macrophages. a-b**: Cytopathic effect on macrophages. **a)** Macrophage culture 14 days p.iso. These cells were mock infected at day 7 p.iso. **b)** Macrophage culture from the same animal as in Fig. 2a 14 days p.iso. These cells were infected at day 7 p.iso. with the SRLV A4 isolate obtained from goat #2, passaged twice in primary macrophage cultures.

Further passaging of these 4 isolates on primary macrophage cultures obtained from SRLV negative goats confirmed the marked differences between these isolates. As illustrated in Figure 2b, isolate #2 showed a strong cytopathic effect on macrophages, supported by a progressive decrease of RT activity upon passaging (data not shown). Isolate #5 showed a similar, albeit less marked phenotype, whereas isolates #3 and #4 were not cytopathogenic and their RT-activity in the supernatants of macrophage cultures increased with every passage (data not shown).

Passaging of these 4 isolates on GSM and LSM cells was unspectacular and only isolate #5 showed clear formation of syncytia with up to 8 nuclei on the fourth passage and only in GSM cells.

### Phylogenetic characterization

**Figure 3:**
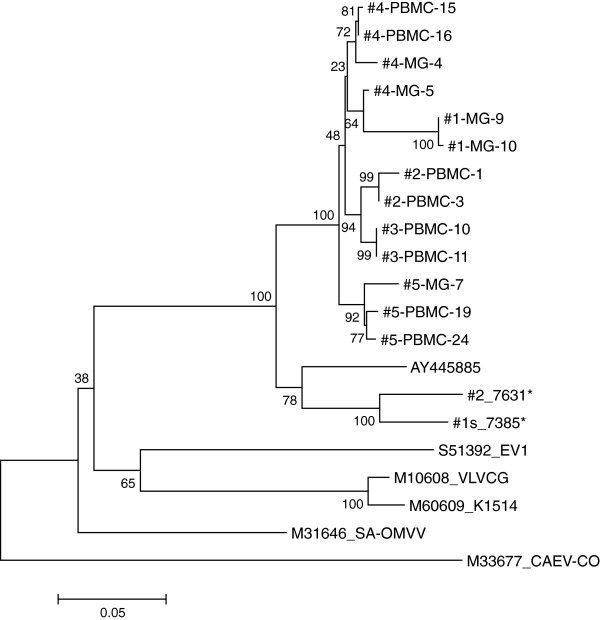
**Phylogenetic analysis.** Phylogenetic analysis of the isolated viruses based on the highly variable SU portion of env encompassing the SU4 and SU5 regions [16,17]. The tree was constructed by the neighbor-joining method, and bootstrap values (1000 replicates) above 70% are considered to be significant. * designates the two SRLV A4 isolates described previously [14].

Several sequences were obtained from PCR fragments amplified from PBMC or mammary gland tissue DNA obtained from the 5 goats. The phylogenetic analysis of these sequences confirmed the serological SU5-ELISA results, classifying the infecting virus as an SRLV A4 subtype. As shown in Figure 3, a phylogenetic analysis based on a highly variable region of the env gene revealed that the SRLV infecting these goats were closely related to each other, although significantly distinct, and that the sequences derived from PBMC or mammary gland of the same animal, where applicable, clustered together. These sequences were closely related to previous Swiss SRLV A4 isolates (#2_7631 and #1s_7385, Figure 3), albeit located on separate branches supported by robust bootstrap values.

### Proviral load in peripheral blood and target organs

The proviral load was quantified by real-time PCR using primers and probes specifically designed on the env gene sequences of these animals. The number of virus copies was calculated by equating the Ct values obtained with the real-time PCR to a standard curve generated based on serial dilutions of a plasmid containing the targeted env region. For every experiment independent standard curves were generated, showing a mean efficiency value close to 100% and a mean R values of R = 0.98.

**Figure 4:**
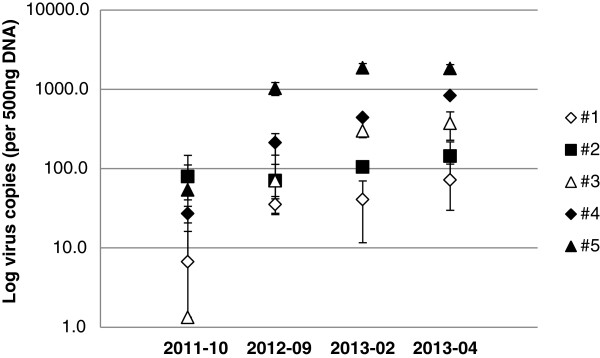
**Monitoring of the proviral load in PBMC.** PBMC associated proviral load was quantified by real time PCR for all 5 goats at 4 time points. Viral loads are indicated as means ± SD.

Successive measurement of the proviral load in PBMC, performed at 4 time points covering 1 ½ year, showed that, with the exception of goat #2, presenting a constant proviral load, the proviral load of the other animals tended to increase over time. This increase was significant (p ≤ 0.05; Bonferroni, All-Pairwise, Multiple Comparison Test) for goat #3, #4 and #5 and strongly suggestive for goat #1 (Figure 4). Goats # 1 and #3 had particularly low proviral loads. At the first sampling time, only 1 out of 3 samples obtained from these goats provided a positive real-time PCR result.

**Figure 5:**
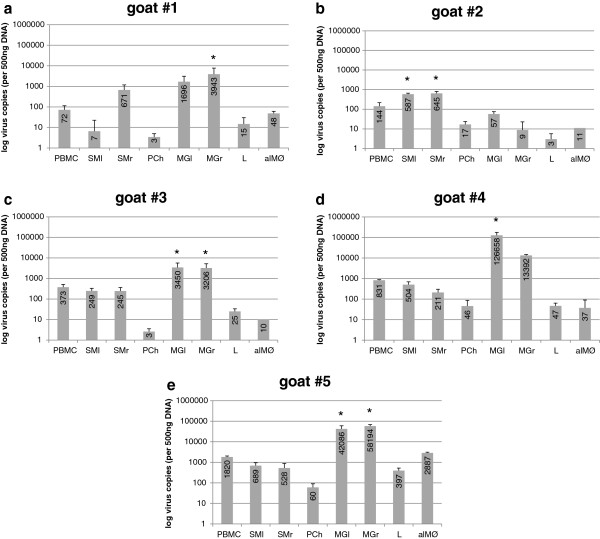
**Quantification of the proviral load in different organs and cells. a-e**: Quantification of the proviral load in different organs and cells. Viral loads of goats #1 to #5 (Figure 5a to 5e) quantified in PBMC, left and right synovial membrane (SMl and SMr), choroid plexus (PCh), left and right mammary glands (MGl and MGr), lung tissue (L) and alveolar macrophages (alMØ). Bars indicate means ± SD and the respective mean value is shown inside the bar. *indicates statistically significant differences according to the Bonferroni (All-Pairwise) Multiple Comparison Test (p ≤ 0.05).

Quantifications of the proviral load in SRLV target organs are shown in Figure 5a-e.

Goat #2 was a clear exception in this group. This animal had a proviral load below 645 copies in all organs tested that, surprisingly, was significantly higher (p ≤ 0.05; Bonferroni, All-Pairwise, Multiple Comparison Test) in the synovial membrane samples. This was not the case for the other 4 goats with a comparable proviral load in synovial membranes and PBMC. In these 4 goats the highest proviral load was detected in the mammary gland. This difference was statistically significant (p ≤ 0.05; Bonferroni, All-Pairwise, Multiple Comparison Test) for the proviral load in the right mammary gland of goat #1 (3943 copies), the left mammary gland of goat #4 (126658 copies) and both half of goat #3 (3450 copies left, 3206 copies right) and #5 (42086 copies left, 58194 copies right). The proviral load in the choroid plexus cells (3 to 60 copies) as well as in lung tissue (3 to 397 copies) was relatively low in all the animals.

### Clinical, pathological and histopathological examination

The animals were monitored by an experienced veterinarian on a biweekly base during the entire experiment. The goats were in an optimal nutrition state, did not show sign of lameness or neurological symptoms and the carpal/metacarpal ratios at the end of the experiment were all in the normal range (< 1.8), i.e.: #1, left (L) 1.53, right (R) 1.62; #2, L 1.57, R 1.55; #3 L 1.51, R 1.57; #4 L 1.54, R 1.5; #5 L 1.47, R 1.48.

**Figure 6:**
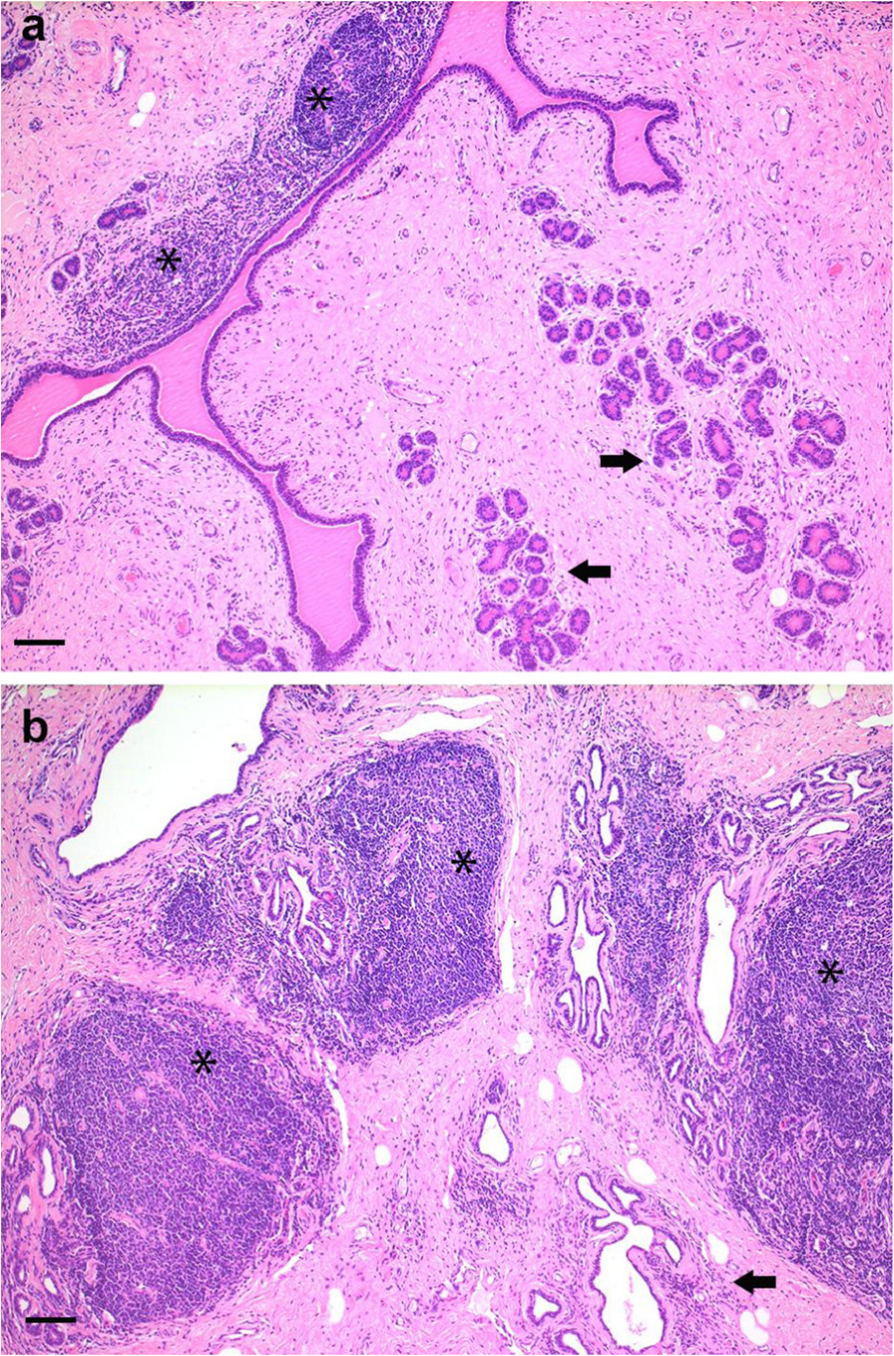
**Histopathology of the mammary gland. a-b**: Histopathology of the mammary gland. **a**: representative histopathological picture of a grade 1 inflammatory reaction in goat #2 with small periductular lymphoid follicles (asterisks) and mild periacinar lymphocytic infiltrates (arrows). **b**: representative histopathological picture of a grade 4 inflammatory reaction in goat #5 with many large lymphoid follicles (asterisks) and moderate lymphocytic periacinar infiltrates (arrow). H&E stain, scale bar = 100 μm.

No gross pathological lesions were observed at necropsy. The histopathological examination of the lung, synovial membranes of joints and choroid plexus did not reveal evidence of inflammatory processes potentially associated with SRLV infections. In the mammary gland of all goats periacinar lymphocytic and plasmacytic infiltrates and extranodal lymphoid follicles adjacent to acini and ducts were present. In goat #2, these changes were very mild (grade 1, Figure 6a), whereas goats #1, #3, and #4 showed more pronounced inflammatory infiltrates and more lymphoid follicles (grade 3). The mammary tissue of goat #5 had the highest histopathological score with large numbers of lymphoid follicles adjacent to acini and ducts (grade 4, Figure 6b).

## Discussion

In spite of a long and expensive CAEV eradication campaign, seropositive goats continue to be detected in Swiss flocks. We have previously shown that the serological tools used to implement this campaign are quite effective at detecting animals infected with classical CAEV strains (SRLV B subtypes), but perform poorly when applied to the detection of goats infected with SRLV A subtypes [14]. The combination of a long eradication campaign, based on the elimination of seropositive animals and their descendants, and the use of serological tools biased toward an efficient detection of SRLV B subtypes most likely favored the spread of SRLV A subtypes in the goat population. In sharp contrast to the situation preceding the CAEV eradication campaign, no clinical cases of SRLV induced pathology such as carpitis, encephalitis or mastitis have been recorded in Switzerland in the last 15 years. This is strong, albeit indirect, epidemiological evidence that for goats the SRLV A subtypes are attenuated. Based on this evidence the eradication campaign is now exclusively focused on goats infected with SRLV B subtypes and SRLV A infected goats are no longer eliminated from the flocks. As a direct consequence of this decision, we are expecting a constant increase in the number of SRLV A infected goats the coming years.

It is therefore crucial to study the SRLV A subtypes currently circulating in Switzerland and to actively search for potential subclinical manifestations of disease induced by these viruses in naturally infected goats. To this purpose we selected a flock of goats infected with the prevalent SRLV A4 subtype from a geographic region epidemiologically not linked to a previously described flock [14]. SRLV infections persist in the infected animals and, with the exception of encephalitis in young goats, the pathological manifestations appears months to years after infection [15]. We selected older animals in order to increase the chances of detecting subclinical manifestations of disease. The absence of clinical signs of arthritis in the entire flock and in these 5 chronically infected adult goats, as demonstrated by their inconspicuous carpal/metacarpal ratio, confirms previous reports on flocks infected with similar viruses.

The phylogenetic characterization of the viruses infecting these 5 goats was based on a region of the env gene encompassing the highly variable SU4 and SU5 domains [16,17]. This permitted us to confirm the SRLV A4 classification of these isolates, anticipated by their SU5 ELISA serological reaction (data not shown) [18]. The four isolates were closely related to previous Swiss A4 viruses but formed a separate cluster supported by strong bootstrap values (Figure 3). The fine phylogenetic resolution permitted by the variability of this env region revealed a coherent grouping of sequences obtained from different anatomical compartments of the same animals, such as PBMC and mammary gland tissue (Figure 3). Taken together, these observations convincingly exclude potential laboratory contaminations in this work.

Virus was readily isolated from 4 out of 5 goats, which points to a potentially high proviral load in the peripheral blood of these animals. The isolated viruses showed marked differences in their in vitro behavior. The isolates from goats #2 and #5 were manifestly cytopathogenic for macrophages, as already observed with a previous A4 isolate obtained from a goat. By contrast, the isolates from goat #3 and #4 were not cytopathogenic for macrophages behaving like SRLV A4 isolated from sheep in a previous work (Figure 2a-b) [14]. Cytopathogenicity appeared to be associated with the replication capacity of these viruses in macrophages, as shown by the rapid increase of RT activity in the supernatant of primary macrophage cultures of goats #2 and #5, measured at day 3 p.iso. (Figure 1). The behavior of these 4 primary isolates on GSM cells was similar to that observed with previous A4 isolates, inducing mild cytopathic effects on these cells. By contrast, these goat isolates did not induce cytopathic effects on LSM cells, as previously observed with an A4 goat isolate inducing large syncytia in LSM [14]. Similarly, SRLV B strains were shown to be severely restricted in their replication in ovine fibroblasts [19]. The lack of cytopathic effects correlated with poor replication on GSM and LSM as demonstrated by testing the supernatants of these cultures by PERT assay, often negative, and with a sensitive RT-PCR assay, generating positive results but with very weak bands (data not shown). Similar observations were reported by Singh and colleagues working with macrophage tropic SRLV isolated from sheep [20]. These authors demonstrated the capacity of infected macrophages to mediate the infection of non-permissive cells such as fibroblasts. In this respect, the preferential homing of infected monocytes to particular tissues, such as the mammary gland, may explain the peculiar pattern of proviral loads observed in our goats.

The final goal of this work was to examine the classical SRLV target organs for their proviral load and the potential presence of histopathological lesions. We have previously shown that there is an excellent correlation between the amounts of genomic RNA and proviral DNA in the peripheral blood and target organs of SRLV infected animals and that the proviral load is a more constant and reliable marker to assess the viral load of infected animals [21].

Proviral load measurements were always performed in triplicates and, for the target organs, on three independent samples obtained from different locations.

With the exception of goat #2, the proviral load in the peripheral blood increased over time showing significant differences between the early time points and the latest sampling times in 3 out of 5 goats. This is a rather unexpected observation, suggesting a progressive loss of control by the immune system that, however, did not result in obvious pathological manifestations. The goats were kept under ideal conditions, excluding stress as a potential trigger for the increasing viral load. Moreover, these animals were adapted to their new environment for a period of 4 months prior to proviral load quantification in PBMC samples. In contrast to previous observations in an SRLV A4 infected, mixed flock of goats and sheep, the proviral loads in the PBMC of these goats were surprisingly high and comparable to those measured in SRLV A4 infected sheep, SRLV B infected goats or HIV infected patients [14,22-24].

The concurrence of a high proviral loads in the absence of overt pathology in the infected animals is reminiscent of the situation observed in SIV infected natural hosts, such as sooty mongabeys and African green monkeys [25]. In both SIV and SRLV the absence of pathological manifestations is probably related to the long co-evolution between these lentiviruses and their respective hosts. In spite of their obvious similarities, such as the sharing of a marked tropism for macrophages, however, primate and small-ruminant lentiviruses are quite distinct. SRLV do not infect lymphocytes and do not induce overt immunosuppression. Therefore, we think that these two lentiviruses adopted different strategies to coexist with their hosts. The so called apathogenic SRLV restricted their expression to organs directly involved in their efficient transmission, such as the mammary gland, while keeping low viral loads in other tissues such as the carpal joints and the choroid plexus. By contrast, apathogenic primate lentiviruses are present at high titers in the entire body but their hosts adapted to the presence of these viruses by modulating the expression of receptors and co-receptors on the surface of target cells such as central memory T cells, which are indispensable to preserving immunocompetence.

The results obtained in target organs and, in particular, in the mammary gland showed small standard deviations between samples taken in the same location and quite large variations between samples taken in different locations of the same organs. This is not surprising and confirms previous observations indicative of a clear compartmentalization of the virus in the target organs [26].

We did not observe an obvious association between the in vitro behavior of these viruses and their proviral load. Indeed, goats #2 and #5, from which the most cytopathogenic viruses were isolated, ranked at the opposite sides of the proviral load scale, with goat #5 showing the overall highest proviral load and #2 the second lowest. Independently of the lactation stage and with the exception of, again, goat #2 the highest proviral load was detected in the mammary gland confirming previous results obtained with goats experimentally infected with an SRLV B molecular clone [21]. These proviral loads were up to 3 orders of magnitude higher than those measured in other tissues, confirming the tropism of these viruses for the mammary gland. In view of the importance of colostrum and milk for the transmission of SRLV this is not surprising. Indeed, the mammary gland was shown to be the only target organ that efficiently permits transmission of highly attenuated SRLV E1 in goat familiar lineages [7,27]. Additionally, we had previously shown that SRLV A and B co-infected mothers tend to transmit the SRLV A subtype more efficiently to their offspring than the B subtype, suggesting that this particular subtypes are particularly efficient in lactogenic transmission [28]. This is in contrast to the situation observed in naturally infected SIV hosts that, in spite of the high viral load and in contrast to HIV infected mothers, do not efficiently transmit SIV to their offspring [25].

The proviral load in the synovial membrane tissue was moderately low and comparable to that observed in PBMC. The lowest proviral load was detected in DNA extracted from choroid plexus cells, suggesting a poor neurotropism for these viruses [12]. Similar proviral loads, albeit at a slightly higher level, were found in the lung and lung macrophages suggesting that these viruses are unlikely to induce lung pathologies.

Gross pathological lesions were not observed and the histopathological findings were also unremarkable. With the exception of the mammary gland, no clear evidence of SRLV induced lesions was found.

In the light of the very high proviral loads detected in the mammary gland, the histopathological examination of this tissue was of particular interest. SRLV infections typically cause diffuse interstitial lympho-plasmacytic infiltrations and formation of lymphoid follicles in the mammary gland [29]. Such histopathological changes were present in all our goats and, interestingly, the different histopathological grades reflected proviral loads in the mammary tissue. The highest number of follicles was found in goat #5, which indeed showed a very high proviral load in the mammary gland tissue (Figure 6b). By contrast, in the mammary tissue of goat #2, which showed a low proviral load, only few lymphoid follicles were observed (Figure 6a). These results, however, should be interpreted with caution, indeed, mild histopathological lesions were observed in goats infected with the highly attenuated SRLV E subtype and also in control age matched animals ( [7,27] and Sergio Rosati personal communication).

## Conclusions

Finally, we conclude that these SRLV A4 viruses are closely related to previous A4 viruses isolated from Swiss flocks. The phylogenetic relationship is even narrower between the 4 isolates obtained from the analysed flock. In spite of this relatively narrow genetic diversity, however, these SRLV behave quite differently *in vitro* as well as *in vivo*. As observed previously with a subtype A4 virus isolated from a goat, 2 isolates were highly cytopathic for macrophages, whereas the other 2 did not show cytopathic effects on these cells. The most important difference compared to a previous study concerned the proviral loads measured *in vivo* that were orders of magnitude higher than those observed in a flock infected with similar viruses. The histopathological examination of different tissues still supports the concept that these particular SRLV are attenuated for goats. Nevertheless, in view of the histopathological lesions detected in the mammary gland and the enormous plasticity demonstrated by these viruses, we are convinced that a continuous and precise monitoring of these SRLV is of utmost importance to avoid the risk of jeopardizing more than 20 years of intensive CAEV eradication efforts.

## Methods

### Animals

Five clinically healthy goats were chosen from a large herd of more than 100 animals according to their serological profile, characterized by high absorbance values in SU5-A4 ELISA [18]. The goats were of different breeds or crossbreeds without pedigree. At the end of the experiment the animals were aged: #1, five; #2, six; #3, eight; #4, seven and #5, six years. At the beginning of the experiment, goats #1 and # 2 were dry does, while the other animals were lactating. The carpal/metacarpal ratio was determined using a measuring tape. Lastly, the animals were euthanized according to Swiss animal welfare regulations by intravenous injection of pentobarbital before necropsy and organ and tissue sampling. These experiments were approved by the committee for animal experimentation of the canton of Bern, licence 98/10.

### PCR primers

Primers were synthesized by Microsynth AG (Balgach, Switzerland). The Minor Groove Binding (MGB) probe was designed with Primer Express® Software 3.0 and synthesized by Applied Biosystems (Life Technologies Europe B.V., Zug, Switzerland).

### PCR protocol

PCR was performed using the HotStarTaq Master Mix Kit (Qiagen AG, Hombrechtikon, Switzerland) in a T3 Thermocycler (Biometra, Göttingen, Germany). The PCR mix with a total volume of 50 μl contains 2.5 units HotStarTaq DNA Polymerase, 1xPCR buffer, 200 μM of each dNTP, 0.5 μM of each primer and 500 ng DNA. For nested or seminested PCR, 5 μl of the amplified PCR product was used for the second round.

PCR Conditions: The hot-start polymerase was activated at 95°C for 15 min followed by 35 or 40 cycles of 94°C for 1 min, annealing temperature for 1 min (see Table 1), 72°C for 1 min and a final extension step of 72°C for 10 min. For the detection of the specific PCR fragments 1.5% or 1% TAE agarose gel containing ethidium bromide was used.

**Table 1:** Primers and probe

**Assay**	**Gene**	**Primer name**	**Sequence**	**Orientation**	Position*	**Product length**	**Annealing temperature**	**PCR round**
Seminested	env	563	GAYATGRYRGARCAYATGAC	Forward	7272-7291	818 bp	47°C	1
PCR		564	GCYAYATGCTGIACCATGGCATA	Reverse	8067-8089	818 bp	47°C	1
		567	GGIACIAAIACWAATTGGAC	Forward	7482-7502	608 bp	49°C	2
		564	GCYAYATGCTGIACCATGGCATA	Reverse	8067-8089	608 bp	49°C	2
PERT assay		MS2-5R	TGTAAGCCTGTGAACGCGAG	Reverse	1464			
		MS2-4f	CCGTATGGCCAACAACTGG	Forward	1214			
		MS2-P45	AGAGCCCTCAACCGGAGTTTGAAGCA	Probe	1310			
Quantitative	env	WasJam-F	ATGCCACAATCCTATATTCAAAATC	Forward	7833-7855			
Real time PCR		WasJam-R	GCTGCCTCTAACACTTGCTG	Reverse	8046-8065			
		WaJaRealTP	TGCTTGCTATCATGGCT	Probe	7927-7943			

Table 1 present a list of the different primers and the probe used for the seminested PCR in the env region, the PERT assay and the quantitative real time PCR.

*bp positions refer to the prototype CAEV-CO molecular clone, GenBank accession number M33677.

### Reverse transcriptase PCR (RT-PCR) protocol

RT-PCR was performed with the OneStep RT-PCR kit (Qiagen AG, Hombrechtikon, Switzerland). Reactions were carried out with a total volume of 50 μl consisting of 1x Qiagen OneStep RT PCR Buffer, 0.4 mM of each dNTP, 0.6 μM of each primer, 10 U RNasin and Qiagen OneStep RT-PCR Enzyme Mix. After a RT step at 50°C for 30 min and an initial polymerase activation step of 95°C for 15 min the following cycling conditions were applied for 40 cycles: 94°C for 30 s, annealing temperature for 1 min, 72°C for 1 min, followed by 72°C for 10 min as a final extension step. For primers and probe sequences see Table 1.

### Quantitative env real-time PCR

This quantitative real-time PCR was developed based on an alignment of env sequences. These were obtained by semi-nested PCR amplification and sequencing of the target region. A reference plasmid was used to generate a standard curve based on 10 fold serial dilutions from 10^6^ to 1 copy.

The following cycling conditions were used: 50°C for 2 min and 95°C for 10 min followed by 40 cycles with 95°C for 15 s and 60°C for 1 min.

The PCR mix contained 1x Maxima Probe qPCR Master Mix (Fermentas GmbH, St. Leon-Rot, Germany), 300 nM each primer, 200 nM probe and 0.15 μl ROX (1:10).

Test- Result: Average of 3 values per template.

### PBMC isolation

Peripheral blood mononuclear cells (PBMCs) were isolated from 20 to 100 ml EDTA- or ACD- anticoagulated blood, by gradient density centrifugation in Ficoll separation solution, as previously described [18]. Cell pellets (5×10^6^ cells/tube) were stored at -80°C.

From PBMCs of 5 animals, macrophages were cultured as previously described in 15% goat serum (Sigma-Aldrich Chemie, GmbH, Buchs, Switzerland) Medium [18].

Cell culture supernatants were collected at day 3, 6, 10 and 13 and frozen at -80°C.

### Tissue samples and histopathological analysis

After euthanasia of the 5 goats, several samples of lung, udder, choroid plexus and synovial membrane were collected for DNA isolation and histopathological analysis.

For DNA isolation tissue samples were quick-frozen on dry ice and stored at -80°C.

For histopathological examination, samples were fixed in 4% buffered formaldehyde, afterwards embedded in paraffin, cut at 5 μm and stained with hematoxylin and eosin. Presence of inflammatory infiltrates in mammary glands was semi-quantitatively graded, assigned grades were: 0 (no infiltrates), 1 (few periacinar lymphyctes/plasma cells and few lymphoid follicles), 2 (moderate amounts of periacinar lymphyctes/plasma cells and few lymphoid follicles), 3 (moderate amounts of periacinar lymphyctes/plasma cells and multiple lymphoid follicles), 4 (moderate amounts of periacinar lymphyctes/plasma cells and many lymphoid follicles), 5 (large amounts of periacinar lymphyctes/plasma cells and many lymphoid follicles).

### Bronchoalveolar lavage (BAL)

From the right lung lobe alveolar macrophages were extracted with a BAL as described previously [18]. Pellets containing 5×10^6^ cells for DNA isolation were stored at -80°C.

### DNA isolation

DNA was extracted from different cell pellets such as PBMC, alveolar macrophages and tissues such as choroid plexus, lung, udder and synovial membrane. In organs DNA was isolated from 3 different locations.

DNA isolation was performed with the DNeasy Blood and Tissue Kit (Qiagen AG, Hombrechtikon, Switzerland) according to the manufacturer’s instructions.

### Cloning and sequencing

PCR products were either directly cloned or first separated on a 1% low melting gel (Nusieve GTG Agarose, Cambrex, Rockland, USA) excised and cloned with Topo TA Cloning kit (Life Technologies Europe B.V. (Invitrogen), Zug, Switzerland) according to manufacturer’s instruction. After transformation into One Shot Top 10 chemically competent E. coli (Life Technologies Europe B.V. (Invitrogen), Zug, Switzerland) bacteria were grown on a LB-amp-X-Gal plate overnight at 37°C. Four to ten colonies were picked and grown overnight at 37°C in LB medium supplemented with 100 μg ampicillin/ml (Ampicillin-sodium salt, Carl Roth GMBH, Karlsruhe, Germany).

Plasmid DNA purification was performed with the QuickLyse Miniprep Kit (Qiagen AG, Hombrechtikon, Switzerland). The sequencing was done by Microsynth (Balgach, Switzerland). Sequences were edited and analyzed with the following software: DNASTAR software (version 5), clone manager, ClustalX 2.0.3. Phylogenetic trees were constructed with Mega (version 5.1) software [30]. Sequence data were submitted to GenBank and were assigned the accession numbers [GenBank: KF990530 to GenBank: KF990540].

### PERT assay

RT activity in the supernatants of infected cells was monitored by PERT assay as described previously [14].

### SRLV replication in cell culture

SRLV positive supernatant of the primary macrophage culture was used to infect SRLV free GSM, LSM and goat macrophages.

SRLV negative GSM, LSM and primary macrophages were seeded in cell culture flasks and left overnight at 37°C and 5% CO_2_ atmosphere. The next day cells were infected with SRLV positive supernatant of primary macrophages. GSM and LSM were then incubated for 10 days and further passaged all 7 to 10 days 3 or 4 times. Infected primary macrophages were incubated for 7 days and the supernatants transferred to freshly, uninfected macrophages every week, for 4 passages. Supernatant of all cell cultures were tested with PERT or a RT-PCR, at each passage. All cell cultures were stained with Diff-Quick® (Medion Grifols Diagnostics AG, Düdingen, Switzerland) at each passage and examined for signs of cytopathic effect (CPE) under a microscope.

### Statistical analysis

All statistical procedures were performed with the NCSS 2007 software (Number Cruncher Statistical Systems, Kaysville, Utah, USA) using Analysis of Variance (one way ANOVA) and Bonferroni (All-Pairwise) Multiple Comparison Test [31].

## Competing interests

The authors declare that they have no competing interests.

## Authors’ contributions

MD preformed the majority of the experiments and co-drafted the manuscript. LB-C participated in the sequence alignment, the design of primers and probe and the provirus quantification. M-LZ contributed to the sample preparations and the analysis of the proviral load. RZ participated in the design of the study and performed the statistical analysis. H-RV participated in the design of the study, performed all the clinical examinations and the necropsy. OP performed all histopathological analyses. GB designed the study and drafted the manuscript. All authors read and approved the final manuscript.

## References

[B1] Bertoni G Blacklaws B Desport M Small ruminant lentiviruses and cross-species transmission Lentiviruses and Macrophages: Molecular and Cellular Interactions 2010 Norfolk, UK: Caister Academic Press 277 306

[B2] Heaton MP Clawson ML Chitko-McKown CG Leymaster KA Smith TP Harhay GP White SN Herrmann-Hoesing LM Mousel MR Lewis GS Kalbfleisch TS Keen JE Laegreid WW Reduced lentivirus susceptibility in sheep with TMEM154 mutations PLoS Genet 2012 8 e1002467 10.1371/journal.pgen.1002467 22291605 PMC3266874

[B3] White SN Mousel MR Herrmann-Hoesing LM Reynolds JO Leymaster KA Neibergs HL Lewis GS Knowles DP Genome-wide association identifies multiple genomic regions associated with susceptibility to and control of ovine lentivirus PLoS ONE 2012 7 e47829 10.1371/journal.pone.0047829 23082221 PMC3474742

[B4] Ruff G Lazary S Evidence for linkage between the caprine leucocyte antigen (CLA) system and susceptibility to CAE virus-induced arthritis in goats Immunogenetics 1988 28 303 309 10.1007/BF00364227 3169879

[B5] Harmache A Russo P Vitu C Guiguen F Mornex JF Pepin M Vigne R Suzan M Replication in goats in vivo of caprine arthritis-encephalitis virus deleted in vif or tat genes: possible use of these deletion mutants as live vaccines AIDS Res Hum Retroviruses 1996 12 409 411 10.1089/aid.1996.12.409 8882321

[B6] Turelli P Guiguen F Mornex JF Vigne R Querat G dUTPase-minus caprine arthritis-encephalitis virus is attenuated for pathogenesis and accumulates G-to-A substitutions J Virol 1997 71 4522 4530 9151845 10.1128/jvi.71.6.4522-4530.1997 PMC191673

[B7] Reina R Grego E Bertolotti L de Meneghi D Rosati S Genome analysis of small-ruminant lentivirus genotype E: a caprine lentivirus with natural deletions of the dUTPase subunit, vpr-like accessory gene, and 70-base-pair repeat of the U3 region J Virol 2009 83 1152 1155 10.1128/JVI.01627-08 18987157 PMC2612395

[B8] Villet S Bouzar BA Morin T Verdier G Legras C Chebloune Y Maedi-visna virus and caprine arthritis encephalitis virus genomes encode a Vpr-like but no Tat protein J Virol 2003 77 9632 9638 10.1128/JVI.77.17.9632-9638.2003 12915575 PMC187391

[B9] Angelopoulou K Brellou GD Greenland T Vlemmas I A novel deletion in the LTR region of a Greek small ruminant lentivirus may be associated with low pathogenicity Virus Res 2006 118 178 184 10.1016/j.virusres.2005.12.010 16446005

[B10] Barros SC Ramos F Duarte M Fagulha T Cruz B Fevereiro M Genomic characterization of a slow/low maedi visna virus Virus Genes 2004 29 199 210 15284480 10.1023/B:VIRU.0000036380.01957.37

[B11] Oskarsson T Hreggvidsdottir HS Agnarsdottir G Matthiasdottir S Ogmundsdottir MH Jonsson SR Georgsson G Ingvarsson S Andresson OS Andresdottir V Duplicated sequence motif in the long terminal repeat of maedi-visna virus extends cell tropism and is associated with neurovirulence J Virol 2007 81 4052 4057 10.1128/JVI.02319-06 17287273 PMC1866131

[B12] Andresdottir V Tang X Agnarsdottir G Andresson OS Georgsson G Skraban R Torsteinsdottir S Rafnar B Benediktsdottir E Matthiasdottir S Arnadóttir S Högnadóttir S Pálsson PA Pétursson G Biological and genetic differences between lung- and brain-derived isolates of maedi-visna virus Virus Genes 1998 16 281 293 10.1023/A:1008030706308 9654682

[B13] Agnarsdottir G Thorsteinsdottir H Oskarsson T Matthiasdottir S St Haflidadottir B Andresson OS Andresdottir V The long terminal repeat is a determinant of cell tropism of maedi-visna virus J Gen Virol 2000 81 1901 1905 10900026 10.1099/0022-1317-81-8-1901

[B14] Cardinaux L Zahno ML Deubelbeiss M Zanoni R Vogt HR Bertoni G Virological and phylogenetic characterization of attenuated small ruminant lentivirus isolates eluding efficient serological detection Vet Microbiol 2013 162 572 581 10.1016/j.vetmic.2012.11.017 23206411

[B15] Blacklaws BA Small ruminant lentiviruses: immunopathogenesis of visna-maedi and caprine arthritis and encephalitis virus Comp Immunol Microbiol Infect Dis 2012 35 259 269 10.1016/j.cimid.2011.12.003 22237012

[B16] Valas S Benoit C Baudry G Perrin G Mamoun RZ Variability and immunogenicity of caprine arthritis-encephalitis virus surface glycoprotein J Virol 2000 74 6178 6185 10.1128/JVI.74.13.6178-6185.2000 10846103 PMC112118

[B17] Bertoni G Hertig C Zahno ML Vogt H-R Dufour S Cordano P Peterhans E Cheevers WP Sonigo P Pancino G B-cell epitopes of the envelope glycoprotein of caprine arthritis-encephalitis virus and antibody response in infected goats J Gen Virol 2000 81 2929 2940 11086124 10.1099/0022-1317-81-12-2929

[B18] Mordasini F Vogt HR Zahno ML Maeschli A Nenci C Zanoni R Peterhans E Bertoni G Analysis of the antibody response to an immunodominant epitope of the envelope glycoprotein of a lentivirus and its diagnostic potential J Clin Microbiol 2006 44 981 991 10.1128/JCM.44.3.981-991.2006 16517887 PMC1393135

[B19] Chebloune Y Sheffer D Karr BM Stephens E Narayan O Restrictive type of replication of ovine/caprine lentiviruses in ovine fibroblast cell cultures Virology 1996 222 21 30 10.1006/viro.1996.0394 8806484

[B20] Singh DK Chebloune Y Mselli-Lakhal L Karr BM Narayan O Ovine lentivirus-infected macrophages mediate productive infection in cell types that are not susceptible to infection with cell-free virus J Gen Virol 1999 80 Pt 6 1437 1444 10374961 10.1099/0022-1317-80-6-1437

[B21] Ravazzolo AP Nenci C Vogt H-R Waldvogel A Obexer-Ruff G Peterhans E Bertoni G Viral load, organ distribution, histopathological lesions, and cytokine mRNA expression in goats infected with a molecular clone of the caprine arthritis encephalitis virus Virology 2006 350 116 127 10.1016/j.virol.2006.02.014 16537085

[B22] Damond F Descamps D Farfara I Telles JN Puyeo S Campa P Lepretre A Matheron S Brun-Vezinet F Simon F Quantification of proviral load of human immunodeficiency virus type 2 subtypes A and B using real-time PCR J Clin Microbiol 2001 39 4264 4268 10.1128/JCM.39.12.4264-4268.2001 11724830 PMC88534

[B23] Clarke JR Gates AJ Coker RJ Douglass JA Williamson JD Mitchell DM HIV-1 proviral DNA copy number in peripheral blood leucocytes and bronchoalveolar lavage cells of AIDS patients Clin Exp Immunol 1994 96 182 186 8187325 10.1111/j.1365-2249.1994.tb06539.x PMC1534888

[B24] Rachid A Croise B Russo P Vignoni M Lacerenza D Rosati S Kuzmak J Valas S Diverse host-virus interactions following caprine arthritis-encephalitis virus infection in sheep and goats J Gen Virol 2013 94 634 642 10.1099/vir.0.044768-0 23197577

[B25] Chahroudi A Bosinger SE Vanderford TH Paiardini M Silvestri G Natural SIV hosts: showing AIDS the door Science 2012 335 1188 1193 10.1126/science.1217550 22403383 PMC3822437

[B26] Pisoni G Moroni P Turin L Bertoni G Compartmentalization of small ruminant lentivirus between blood and colostrum in infected goats Virology 2007 369 119 130 10.1016/j.virol.2007.06.021 17719071

[B27] Reina R Juganaru MM Profiti M Cascio P Cerruti F Bertolotti L De MD Amorena B Rosati S Immunological parameters in goats experimentally infected with SRLV genotype E, strain Roccaverano Vet Immunol Immunopathol 2011 139 237 244 10.1016/j.vetimm.2010.11.001 21122927

[B28] Pisoni G Bertoni G Manarolla G Vogt HR Scaccabarozzi L Locatelli C Moroni P Genetic analysis of small ruminant lentiviruses following lactogenic transmission Virology 2010 407 91 99 10.1016/j.virol.2010.08.004 20797752

[B29] Jubb KVF Kennedy PC Palmer N Pathology of Domestic Animals 2007 Edinburgh: Elsevier Saunders

[B30] Tamura K Peterson D Peterson N Stecher G Nei M Kumar S MEGA5: molecular evolutionary genetics analysis using maximum likelihood, evolutionary distance, and maximum parsimony methods Mol Biol Evol 2011 28 2731 2739 10.1093/molbev/msr121 21546353 PMC3203626

[B31] Sokal RR Rohlf FJ Biometry: The Principles and Practice of Statistics in Biological Research 1995 New York: W.H. Freeman and Company

